# Complex Crystal Structure Determination and *in vitro* Anti–non–small Cell Lung Cancer Activity of Hsp90^*N*^ Inhibitor SNX-2112

**DOI:** 10.3389/fcell.2021.650106

**Published:** 2021-03-29

**Authors:** Dong Zhao, Yi-Ming Xu, Lu-Qi Cao, Feng Yu, Huan Zhou, Wei Qin, Hui-Jin Li, Chun-Xia He, Lu Xing, Xin Zhou, Peng-Quan Li, Xin Jin, Yuan He, Jian-Hua He, Hui-Ling Cao

**Affiliations:** ^1^Shaanxi Key Laboratory of Ischemic Cardiovascular Disease, Institute of Basic & Translational Medicine, Xi’an Medical University, Xi’an, China; ^2^Department of Medicinal Chemistry, Virginia Commonwealth University, Richmond, VA, United States; ^3^Shanghai Synchrotron Radiation Facility, Shanghai Advanced Research Institute, Chinese Academy of Sciences, Shanghai, China; ^4^College of Chemistry and Materials Science, Key Laboratory of Synthetic and Natural Functional Molecule, Ministry of Education, Northwest University, Xi’an, China; ^5^Institute for Advanced Studies, Wuhan University, Wuhan, China

**Keywords:** heat shock protein 90^*N*^, non–small-cell lung cancer, drug development, SNX-2112, complex crystal structure, molecular interaction

## Abstract

SNX-2112, as a promising anticancer lead compound targeting heat shock protein 90 (Hsp90), absence of complex crystal structure of Hsp90^*N*^-SNX-2112 hindered further structural optimization and understanding on molecular interaction mechanism. Herein, a high-resolution complex crystal structure of Hsp90^*N*^-SNX-2112 was successfully determined by X-ray diffraction, resolution limit, 2.14 Å, PDB ID 6LTK, and their molecular interaction was analyzed in detail, which suggested that SNX-2112 was well accommodated in the ATP-binding pocket to disable molecular chaperone activity of Hsp90, therefore exhibiting favorable inhibiting activity on three non–small cell lung cancer (NSCLC) cell lines (IC_50_, 0.50 ± 0.01 μM for A549, 1.14 ± 1.11 μM for H1299, 2.36 ± 0.82 μM for H1975) by inhibited proliferation, induced cell cycle arrest, and aggravated cell apoptosis. SNX-2112 exhibited high affinity and beneficial thermodynamic changes during the binding process with its target Hsp90^*N*^ confirmed by thermal shift assay (TSA, ΔTm, and −9.51 ± 1.00°C) and isothermal titration calorimetry (*K*_*d*_, 14.10 ± 1.60 nM). Based on the complex crystal structure and molecular interaction analysis, 32 novel SNX-2112 derivatives were designed, and 25 new ones displayed increased binding force with the target Hsp90^*N*^ verified by molecular docking evaluation. The results would provide new references and guides for anti-NSCLC new drug development based on the lead compound SNX-2112.

## Introduction

Lung cancer, as the leading cause of cancer death worldwide, accounts for 18% of total cancer deaths each year (Petrek and Yu, 2019). According to histological types and therapeutic aims, lung cancer can be traditionally classified into small cell lung cancer (SCLC) and non–small cell lung cancer (NSCLC); NSCLC accounts for 80–85% (Ettinger et al., 2017). NSCLC is increasingly thought as an oncogene addicted tumor (Molina et al., 2008); therefore, targeted drugs may be an effective way for NSCLC treatment (Tomasini et al., 2016; Xiong et al., 2019).

Heat shock protein 90 (Hsp90), as an evolutionarily conserved chaperone in eukaryotes, plays a vital role in cell differentiation, proliferation, and apoptosis by assisting more than 300 client protein proper folding or conformational maturation (Li et al., 2012a; Vahid et al., 2017). Among the client proteins, 48 proteins play an important, even decisive role in tumorigenesis and development, such as transmembrane tyrosine kinase, steroid receptors, and cell cycle regulators, etc. (Jhaveri et al., 2014; Hyun et al., 2018). Hsp90^*N*^ inhibitors lead to multiple misfolded or immature client proteins to be degraded by the ubiquitin protease complex pathway (Chen et al., 2014), which involves simultaneous intervention of multiple potential antitumor target proteins and a “multipoint attack” on the tumor to achieve favorable therapeutic efficacy (Chehab et al., 2015; Dutta Gupta et al., 2019). Hsp90 inhibitor development, therefore, has become a promising strategy in cancer therapy (Barrott and Haystead, 2013; Soga et al., 2013; Hu et al., 2019).

Heat shock protein 90 contains four isoforms (Hsp90α, Hsp90β, Trap1, and Grp94), and in cytosol Hsp90 exists in α-α or β-β homologous dimer form, and every monomer consists of three domains (Hsp90^*N*^, Hsp90^*M*^, and Hsp90^*C*^; Donnelly and Blagg, 2008; Mayer et al., 2012). Hsp90 inhibitors accordingly are classified as Hsp90^*N*^ inhibitors, Hsp90^*M*^ inhibitors, and Hsp90^*C*^ inhibitors (Sidera and Patsavoudi, 2014). Hsp90^*N*^ inhibitor development targeting to ATP-binding pocket of Hsp90^*N*^ remains a research hot spot (Prodromou et al., 2000; Li et al., 2012b; Sung et al., 2016). There are four groups for Hsp90^*N*^ inhibitors based on chemical structures including ansamycin groups, resorcinol groups, benzamide groups, and purine groups (Mollapour et al., 2011). On behalf of benzamide groups, SNX-2112 (IC_50_, 3 nM) and its oral form SNX-5422 (IC_50_, 32 nM) displayed high binding affinity with Hsp90^*N*^ and favorable antitumor potency in multiple cancer cell lines (multiple myeloma, melanoma, esophageal cancer, breast cancer, and hepatoma cells; Okawa et al., 2009; Bachleitner-Hofmann et al., 2011; Wang et al., 2014; Fernandes and Alves, 2017; Tabata et al., 2020) and xenograft mouse models (Huang et al., 2009).

So far, there is rare report concerning anti-NSCLC activity of SNX-2112 (Hendriks and Dingemans, 2017). Our preliminary experimental results indicated that SNX-2112 exhibited favorable inhibition activity on NSCLC cell lines (A549, H1299, and H1975). However, absence of the complex crystal structure of Hsp90^*N*^-SNX-2112 hampered further structural optimization of SNX-2112 and understanding of binding mode and molecular interaction of Hsp90^*N*^-SNX-2112. Herein, the complex crystal structure of Hsp90^*N*^-SNX-2112 was determined by X-ray diffraction (XRD); molecular interaction analysis based on complex crystal structure, thermal shift assay (TSA), and isothermal titration calorimetry (ITC) was performed. Meanwhile, *in vitro* anti-NSCLC activity of SNX-2112 was evaluated, which would provide new references for anti-NSCLC new drug development based on the lead compound SNX-2112.

## Materials and Methods

### Materials and Reagents

A549, H1299, and H1975 NSCLC cell lines were obtained from the AOLUKEJI (Shanghai, China). Fetal bovine serum and RPMI 1640 medium were purchased from Gibco-BRL (Gaithersburg, MD, United States).

SNX-2112 was acquired from Target Molecule Corp. (Boston, MA, United States). Dimethyl sulfoxide (DMSO), magnesium chloride (MgCl_2_), PEG4000, sodium acetate (NaAc), Tris, and Tris–HCl and were purchased from Sigma–Aldrich Corp. (St. Louis, MO, United States). Glycerol, ethyl alcohol, sodium hydroxide, hydrochloric acid (HCl), and sodium choride (NaCl) were obtained from Xi’an Chemical Reagent Company (Xi’an, China). SNX-2112 was dissolved in DMSO, and other reagents were dissolved in double distilled water (ddH_2_O).

### Methods

#### Anticancer Activity *in vitro*

##### Cell viability assay

Non–small cell lung cancer cell lines A549, H1299, and H1975 were cultured in RPMI 1640 medium supplemented with 10% (vol/vol) fetal bovine serum at 37°C in a humidified 5% CO_2_ atmosphere incubator (Thermo Fisher Scientific Inc., Waltham, MA, United States). Cell lines were placed in 96-well plates at 4 × 10^3^ per well in triplicates and SNX-2112 (100, 10, 1, 0.1, 0.01 μM) treated cell lines for 72 h (quantitative IC_50_ value). Cell lines were placed in triplicate at 4 × 10^3^ per well in 96-well plates and then treated by SNX-2112 (4 × 0.50 μM for A549, 4 × 1.14 μM for H1299, and 4 × 2.36 μM for H1975) or DMSO for 24, 48, or 72 h (cell viability assay); 2.5 h after adding CCK-8, OD_450_ was detected by CCK-8 assay (7Sea, Shanghai, China) using an ELISA plate reader (BioTek Instruments, United States; Xue et al., 2016; Wei et al., 2018).

##### Cell cycle assay

Non–small cell lung cancer cell lines A549, H1299, and H1975 were seeded in a 6-well culture plate at density 3 × 10^5^ cells per well, which were treated with different concentrations of SNX-2112 or DMSO for 72 h (4 × 0.50 μM for A549, 4 × 1.14 μM for H1299, and 4 × 2.36 μM for H1975). Cells were then gathered and washed with cold phosphate-buffered saline (PBS). And then at 4°C, 1 mL PBS and 2 mL 100% ethanol were was added. After centrifugation at 1,000 *g* for 5 min, they were washed with 2 mL PBS and resuspended with 400 μL PBS. In the end, 50 μL propidium iodide (PI) and 50 μL RNase were added, and cells were darkly cultivated for 30 min at 37°C. The effects of SNX-2112 on cell cycle distribution were analyzed with flow cytometry (Becton Dickinson, Franklin Lakes, NJ, United States; Olszewska et al., 2020)

##### Cell apoptosis assay

Based on instructions from the manufacturer, the apoptosis rate was detected by the annexin V–fluorescein isothiocyanate (FITC)/PI Apoptosis Detection kit (556547, Becton Dickinson, United States). Cells were treated identically with mentioned as *Cell Cycle Analysis*. And then, cells were gathered and washed with 2 mL PBS and resuspended in 100 μL binding buffer. Lastly, 5 μL annexin V–FITC and 10 μL PI were added to the buffer and darkly cultivated at 4°C for 30 min. Effects of SNX-2112 on cell apoptosis were determined by flow cytometry (Becton Dickinson, Franklin Lakes, NJ, United States; Ye et al., 2017).

#### Molecular Interaction Analysis

##### Thermal shift assay

Thermal shift assay was applied for evaluating molecular interaction between target Hsp90^*N*^ and its ligand SNX-2112 using real-time PCR (7500, ABI Corp., United States). A 20-μL reaction system consisted of Hsp90^*N*^ (1 mg/mL) 2 μL, ligand SNX-2112 (dissolved in DMSO, 100 mM) 0.5 μL, buffer (pH 7.5, 20 mM Tris–HCl, 150 mM NaCl, and 10% glycerol) 10 μL, Protein Thermal Shift Buffer (Applied Biosystems, United States) 5 μL, and TSA dye (Applied Biosystems, United States) 2.5 μL, eight replicates. The samples were centrifuged at 1,000 revolutions/min (rpm) for 1 min. The samples were run from 25 to 95°C with a ramp rate of 1°C/min. The protein unfolds when it is heated and exposes hydrophobic regions to bind the environmentally sensitive TSA dye and fluoresces. The melting temperature (Tm) and melting temperature differences (ΔTm) were derived from the melting curve, which was related to the binding affinity of Hsp90^*N*^-SNX-2112 (Andreotti et al., 2015).

##### ITC

Isothermal titration calorimetry was used for further detecting Hsp90^*N*^-SNX-2112 molecular interactions using ITC (ITC-200, Malvern Instrument Ltd., United Kingdom). SNX-2112 in DMSO was diluted with buffer for protein purification (pH 7.5, 20 mM Tris–HCl and 150 mM NaCl) to 500 μM. Fresh-purified Hsp90 was extensively dialyzed against the same buffer and concentrated to 50 μM. After being centrifuged and degassed, 2 μL aliquots of SNX-2112 were injected into Hsp90^*N*^ solution in the cell with an interval of 200 s and 750-rpm stirring speed. With Microcal Origin software, the experimental data were fitted with a bimolecular binding model with stoichiometry (*n*), enthalpy (Δ*H*°), and association constant (*K*_*a*_) as adjustable parameters. The thermodynamic parameters Δ*G*° (free energy) and Δ*S*° (entropy) were derived from the equation -*RT* ln *K*_*a*_ = Δ*G*°= Δ*H*° - *T*Δ*S* (He et al., 2014b).

#### Complex Crystal Structure Determination

*Homo sapiens* Hsp90^*N*^ gene containing residues 9 to 236 was artificially synthesized and cloned into plasmid pET-28a, which was transformed into *Escherichia coli* BL21 (DE3; TIANGEN Biotech Corp., Beijing, China) to express and purify Hsp90^*N*^ as reported by Cao (Cao et al., 2017).

With 5:1 molar ratio, SNX-2112 was added into Hsp90^*N*^ to incubate for 30 min at 4°C. Then, the mixture was centrifuged for 10 min at 3,000 *g*, and the supernatant was taken to mix with the same amount of a crystal precipitant [pH 8.5, 100 mM Tris–HCl, 200 mM MgCl_2_, 25–30% PEG4000 (Stebbins et al., 1997)]. With the hanging drop vapor diffusion method, cocrystallization was carried out at 4°C for 3–7 days in an incubator controlled by a bath circulator (PolyScience 9712, PolyScience, United States). Complex crystal image of Hsp90^*N*^-SNX-2112 was captured by a stereomicroscope (M165, Leica Microsystems, Germany).

Complex crystals were mounted with cryo-loop (Hampton Research Corp., Aliso Viejo, CA, United States), and then quickly soaked in the cryoprotectant solution containing 20–25% glycerol and crystal reagent mentioned previously (Prodromou et al., 2000). Then, crystals were flash-frozen in liquid nitrogen for XRD. With an ADSC Quantum 315r CCD detector (Wang et al., 2015), all data sets were collected at 100 K on Macromolecular Crystallography Beamline17U1 (BL17U1) at Shanghai Synchrotron Radiation Facility (SSRF, Shanghai, China; Wang et al., 2018).

Aquarium pipeline was applied for automatic processing of diffraction data (Yu et al., 2019b). Using the crystal structure of Hsp90^*N*^-FS23 (PDB ID 5CF0) as the research model (Li et al., 2015), the complex crystal structure was determined and refined by molecular replacement with PHENIX software (Adams et al., 2010; Yu et al., 2019a). The initial model was rebuilt by Coot software (Emsley et al., 2010), and CCP4MG software was applied for graphing and molecular interaction analysis (McNicholas et al., 2011; Winn et al., 2011).

#### Design of New SNX-2112 Derivatives and Molecular Docking Evaluation

A series of new SNX-2112 derivatives were designed based on the complex crystal structure and molecular interaction analysis, which were evaluated by molecular docking with software SYBYL-X 2.0 (Tripos Associates Inc., St. Louis, MO, United States). The crystal structure of Hsp90^*N*^-FS23 (PDB ID 5CF0) was as the docking model. First, a compound library of newly designed SNX-2112 derivatives was built, and new derivatives were conducted with geometric and force field optimization for 10,000–100,000 times. Second, the target Hsp90^*N*^ was optimized by adding hydrogen and charges. Last, molecular docking was carried out using the Surflex-Dock module of SYBYL software. Total score and Cscore were chosen as parameters to evaluate the binding affinity between the target Hsp90 and new ligands. CCP4MG software was applied for simulated complex three-dimensional (3D) structure reconstruction and molecular interaction analysis.

#### Statistical Analysis

Statistical analysis was conducted with SPSS 13.0 software (International Business Machines Corporation, United States) and GraphPad Prism 5.01 software (GraphPad Software, San Diego, United States). Data were presented as mean ± standard deviation (SD). Differences between two groups were performed according to unpaired Student *t* test. *P* < 0.05 was deemed as statistically significant.

## Results

### Anticancer Activity *in vitro*

#### SNX-2112 Inhibited NSCLC Cell Proliferation

SNX-2112 exhibited a creditable anti-NSCLC activity *in vitro* against A549 cells (IC_50_, 0.50 ± 0.01 μM), H1299 cells (IC_50_, 1.14 ± 1.11 μM), and H1975 (IC_50_, 2.36 ± 0.82 μM).

**FIGURE 1:**
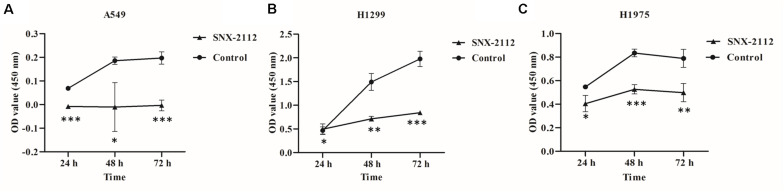
SNX-2112 inhibited NSCLC cell proliferation. **(A)** A549 cells, **(B)** H1299 cells, and **(C)** H1975 cells. CCK-8 assay was performed to detect cell viability after treatment with SNX-2112 or DMSO for 24, 48, and 72 h. *p < 0.05, **p < 0.01, and ***p < 0.001 indicate significant differences vs. control. SNX-2112 significantly suppressed cell viability against A549, H1299, and H1975 cells, and the inhibition activity increased with treatment time extension. Annotations: Control, cells treated with DMSO; SNX-2112, cells treated with SNX-2112.

As shown in Figure 1, SNX-2112 significantly suppressed cell proliferation of A549, H1299, and H1975 at 24, 48, and 72 h (*p* < 0.05), especially for A549 at 24 and 72 h (*p* < 0.001), H1299 at 72 h (*p* < 0.001), and H1975 at 48 h (*p* < 0.001). The results suggested the cell viability of A549, H1299, and H1975 was inhibited by SNX-2112, and the inhibition activity increased with treatment time extension.

#### SNX-2112 Induced NSCLC Cell Cycle Arrest

**FIGURE 2:**
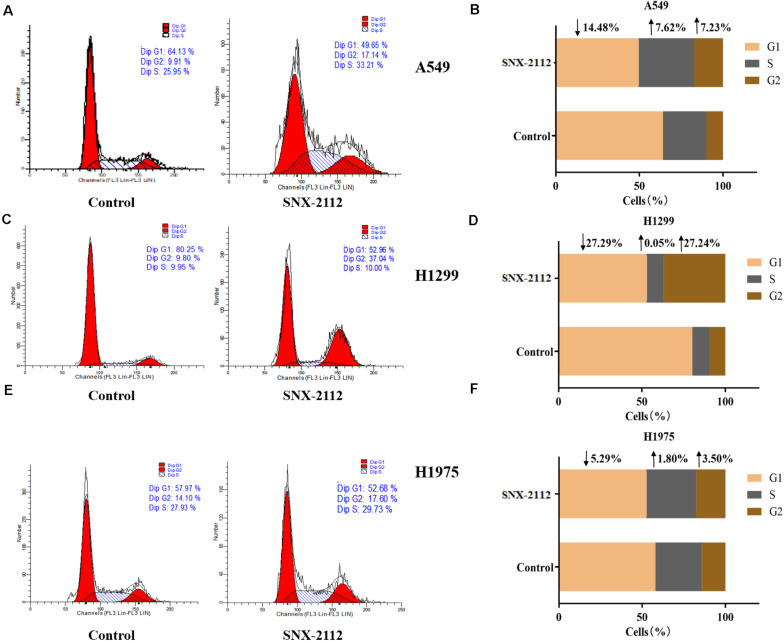
SNX-2112 induced NSCLC cell cycle arrest. Cell cycle distribution for A549 cells **(A)**, H1299 cells **(C)**, and H1975 cells **(E)** treated with SNX-2112 or DMSO for 72 h detected by DNA flow cytometry assay. The DNA content in different phase of A549 cells **(B)**, H1299 cells **(D)**, and H1975 cells **(F)** treated with SNX-2112 or DMSO for 72 h. SNX-2112 induced A549, H1975, and H1299 cells cycle arrest at S and G2 phase or G2 phase. Annotations: Control, cells treated with DMSO for 72 h; SNX-2112, cells treated with SNX-2112 for 72 h.

Flow cytometry was used to explore effects of SNX-2112 on NSCLC cell cycle distribution by calculating cellular DNA content. As can be seen from Figure 2, with treatment with SNX-2112 for 72 h, cell percentage in G1, S, and G2 phase exhibited obvious changes compared with the control treated with DMSO for A549 cells (G1, ↓14.48%; S, ↑7.62%; and G2, ↑7.23% vs. control), for H1299 (G1, ↓27.29%; S, ↑0.05%; and G2, ↑27.24% vs. control), and for H1975 cells (G1, ↓5.29%; S, ↑1.80%; and G2, ↑3.50% vs. control). It was suggested that Hsp90^*N*^ inhibitor SNX-2112 induced cell cycle arrest for A549 and H1975 at G2 and S phase and for H1299 at G2 phase.

#### SNX-2112 Aggravated NSCLC Cell Apoptosis

**FIGURE 3:**
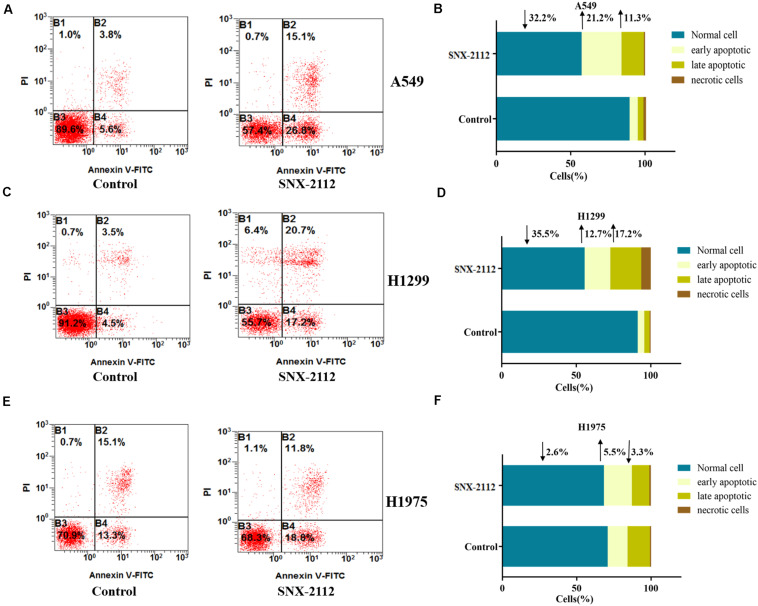
SNX-2112 aggravated NSCLC cell apoptosis. SNX-2112 aggravated apoptosis of A549 cells **(A)**, H1299 cells **(C)**, and H1975 cells **(E)**. Apoptosis determined by flow cytometry method for cells treated with SNX-2112 or DMSO. Distribution difference of A549 cells **(B)**, H1299 cells **(D)**, and H1975 cells **(F)** in four quadrants. The four quadrants represent as B3, Normal cell; B4, early apoptosis; B2, late apoptosis; and B1, necrotic cells. It was revealed that SNX-2112 aggravated NSCLC cell apoptosis. Annotations: Control, treated with DMSO for 72 h; SNX-2112, treated with SNX-2112 for 72 h.

During early apoptosis stage, annexin V combined with phosphatidylserine on the external leaflet of plasma membrane, which excluded PI and stained negatively. However, cells of late apoptosis stage and necrosis stained positively. Figure 3 showed the percentage of specific cell population at different apoptosis stages (B3 for normal cells, B4 for early apoptosis cells, B2 for late apoptosis cells, and B1 for necrotic cells). Compared with the control, SNX-2112 increased apoptotic cell percentage of the three NSCLC cell for A549 (normal cells, ↓32.2%; early apoptosis, ↑21.2%; late apoptosis, and ↑11.3% vs. control), H1299 (normal cells, ↓35.5%; early apoptotic, ↑12.7%; late apoptotic, ↑17.2% vs. control), and H1975 (normal cells, ↓2.6%; early apoptosis, ↑5.5%; late apoptosis, ↓3.3% vs. control). It was indicated that SNX-2112 aggravated cell apoptosis of A549, H1299, and H1975 cells.

To sum up, SNX-2112 disclosed favorable *in vitro* anti-NSCLC activity (IC_50_, 0.50 ± 0.01 μM for A549; 1.14 ± 1.11 μM for H1299; and 2.36 ± 0.82 μM for H1975), which was achieved by inhibiting cell viability, inducing cell cycle arrest, and aggravating cell apoptosis. Therefore, SNX-2112 would be a potential lead compound for anti-NSCLC new drug development.

### Molecular Interaction Analysis

#### TSA

**FIGURE 4:**
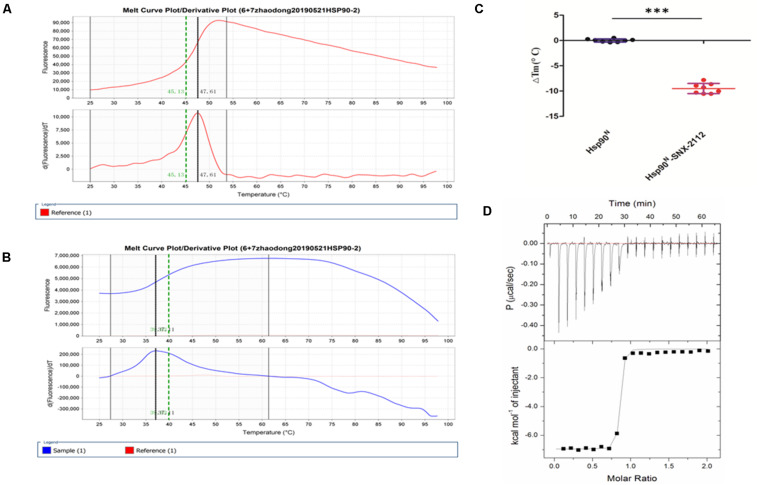
Molecular interaction analysis performed by thermal shift assay (TSA) and isothermal titration calorimetry (ITC). **(A)** Melting curve of Hsp90^*N*^. **(B)** Melting curve of SNX-2112 binding with Hsp90^*N*^. **(C)** ΔTm (°C) value of SNX-2112 binding with Hsp90^*N*^ (−9.51 ± 1.00°C, ***p < 0.001 vs. control, Hsp90^*N*^, *n* = 8). **(D)** ITC plots for the ligand SNX-2112 binding with its target Hsp90^*N*^. The upper panel manifests the raw titration data. The power was supplied to the system to maintain a constant temperature against time (the area of each peak gives the heat of interaction for that injection). The lower panel exhibits the bimolecular fit of the normalized heats of interaction plotted against the molar concentration. Titrations were performed at 25°C in PBS (pH 7.5, 20 mM Tris–HCl, 150 mM NaCl). The values were presented as mean ± SD. The result indicated an intense binding force and favorable thermodynamic changes during the Hsp90^*N*^-SNX-2112 binding process.

Usually, every protein has a specific Tm value under certain conditions, which would change when binding a ligand. Greater ΔTm means higher affinity, and absolute ΔTm value greater than three is deemed as a favorable ligand (Lo et al., 2004; Andreotti et al., 2015). As can be seen from Figure 4, Tm value of Hsp90^*N*^ was 48.36 ± 0.82°C (Figure 4A), and Tm was shifted to 38.84 ± 1.35°C after Hsp90^*N*^ combining with SNX-2112 (Figure 4B). As a result, ΔTm value was -9.51 ± 1.00°C (^∗∗∗^*p* < 0.001 vs. control, Hsp90^*N*^, *n* = 8; Figure 4C). It suggested that an intense binding force existed between the ligand SNX-2112 and its target Hsp90^*N*^.

#### ITC

As shown in Figure 4D, the upper panel manifested the raw titration data. The power was supplied to the system to maintain a constant temperature against time, and the area of each peak meant interaction heat for injection. The lower panel exhibited bimolecular fit of normalized interaction heats plotted against molar concentration. Hsp90^*N*^ binding with SNX-2112 was exothermic at 25°C from the negative peaks in the raw ITC titration data. A fine S-shaped curve was obtained after nonlinear data fitting. The stoichiometry was determined to be 0.83 ± 0.01, indicating a 1:1 binding mode between target Hsp90^*N*^ and ligand SNX-2112. As shown in Table 1, the *K*_*d*_ values here were 14.10 ± 1.60 nM, which indicated an intense binding force between the ligand SNX-2112 and its target Hsp90^*N*^. The thermodynamic signature was dominated by both favorable enthalpy change (Δ*H*_*a*_, −6.80 ± 0.20 kcal/mol) and entropy change (*T*Δ*S*_*a*_, 3.90 kcal/mol), indicating the establishment of favorable interactions during the Hsp90^*N*^-SNX-2112 binding process (He et al., 2011, 2014a).

**TABLE 1:** Dissociation constant and thermodynamic data during the Hsp90^*N*^-SNX-2112 binding process at 25°C.

Items	Thermodynamic data (mean ± SD, *n* = 4)
*n*	0.83 ± 0.01
*K*_*d*_ (nM)	14.10 ± 1.60
Δ*G*_*a*_ (kcal/mol)	−10.70
Δ*H*_*a*_ (kcal/mol)	−6.80 ± 0.20
*T*Δ*S*_*a*_ (kcal/mol)	3.90

### Complex Crystal Structure Determination

With a molecular weight of 25 kDa, Hsp90^*N*^ was expressed in *E. coli* BL21 (DE3) strain. Purified Hsp90^*N*^ protein was obtained by immobilized Ni^++^ affinity chromatography and molecular sieve chromatography. Complex crystals of Hsp90^*N*^-SNX-2112 were obtained by the hanging-drop method at 4°C for 3–5 days. As shown in Supplementary Image 1, the average dimension of rhombus crystals was approximately 230 μm × 130 μm × 50 μm.

Crystal structure of Hsp90^*N*^-FS23 (PDB ID 5CF0) was reported as the research model; a complex crystal structure of Hsp90^*N*^-SNX-2112 was successfully determined by molecular replacement. The complete data collection and refinement statistics are shown in Table 2. The coordinates and structure factors have been deposited in PDB (PDB ID 6LTK). Diffraction data were collected up to 2.14 Å resolution limit with *R*_*free*_ = 0.24 and *R*_*work*_ = 0.21. The space groups showed *I*222 (unit cell parameter, *a* = 69.70 Å, *b* = 88.87 Å, *c* = 96.55 Å; α = β = γ = 90.00°).

**TABLE 2:** Data collection and refinement statistics of Hsp90^*N*^-SNX-2112 complex crystal.

Diffraction source	BL17U1, SSRF^a^
*Diffraction data*	
Resolution (Å)	50.00–2.14 (2.18–2.14)^*a*^
Space group	*I*222
Unit cell parameters	
*a*, *b*, *c* (Å)	69.700 88.868 96.550
α, β, γ (I)	90.000 90.000 90.00
Wavelength (Å)	0.97890
Total reflections	217137
Unique reflections	16764
Redundancy	13.0 (11.4)^*a*^
Mean I/*σ* (I)	90.0/2.6 (2.3/1.8)
Completeness (%)	99.1 (99.1)^*a*^
*R*_*sym*_ *or R*_*merge*_^*a*^	0.074 (1.267)^*a*^
*Refinement data*	
Resolution range (Å)	36.24–2.14
Reflections in working set	15070
Reflections in test set	1674
*R*_*work*_^*b*^/*R*_*free*_^*c*^ (%)	0.21/0.24
Mean temperature factor (Å^2^)	58.2
Bond lengths (Å)	0.002
Bond angles (°)	0.587
Wilson B-factor (Å^2^)	51.5
Total number of atoms	1715
R.m.s. deviations	
Bond lengths (Å)	0.004
Bond angles (°)	0.707
Ramachandran plot	
Favored (%)	97.09
Allowed (%)	2.91
Outliers (%)	0
The temperature factors (*B* values, Å^2^)	57.3274

*^*a*^The numbers in parentheses represent the values for the highest-resolution shells.*

*^*b*^*R*_*work*_ = Σ_*hkl*_*| | F*_*obs*_*|* − | *F*_*calc*_| *|* /Σ_*hkl*_*| F*_*obs*_|, where *F*_*obs*_ and *F*_*calc*_ are observed and calculated structure factors, respectively.*

*^*c*^*R*_*free*_, calculated the same as *R*_*work*_, but from a test set containing 5% of data excluded from the refinement calculation.*

**FIGURE 5:**
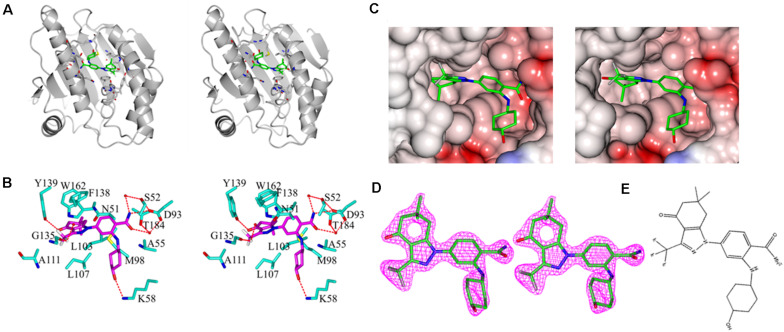
Stereo images of complex crystal structure of Hsp90^*N*^-SNX-2112. **(A)** Overall structure of Hsp90^*N*^-SNX-2112. Hsp90^*N*^ and SNX-2112 are shown as cartoon and stick, respectively. **(B)** Molecular interaction between SNX-2112 and Hsp90^*N*^. The carbon, nitrogen, oxygen, and fluorine atoms of SNX-2112 are colored in magenta, blue, red, and white, respectively. In addition, the carbon, nitrogen, oxygen, and sulfur atoms of Hsp90^*N*^ residues are shown in cyan, blue, red, and yellow, respectively. The hydrogen bonds are represented as red dashed lines. The red balls are on behalf of water molecules. **(C)** Electrostatic potential surface distribution of complex crystal structure of Hsp90^*N*^-SNX-2112. The crystal structure surface is colored to reflect the electrostatic potential: red for negative charge and blue for positive charge. The carbon, nitrogen, oxygen, and fluorine atoms of SNX-2112 are colored in green, blue, red, and gray, respectively. **(D)** Electron density map (2*Fo*-*Fc*) of SNX-2112. SNX-2112 is shown as stick. **(E)** Chemical structure of SNX-2112.

As shown in Figure 5, the asymmetric unit of the refined model included a protein monomer (residues 17–224) and 47 water molecules, as well as one SNX-2112 molecule in the ATP-binding pocket. No electron density was observed for residues 9–16 and residues 225–236 of *N*- and *C*- terminal protein, which was attributed to disordered electron density. The refinement results showed that continuous and strong electron density appears in the empty space of ATP-binding site of Hsp90^*N*^ with a shape to well accommodate SNX-2112 molecule. SNX-2112 was perfectly sitting at the ATP-binding pocket to disable molecular chaperone activity of Hsp90 by blocking ATP binding, therefore suppressing cancer cells.

As shown in Figure 5B, various interactions, including hydrogen bonds, π-π interactions, and hydrophobic interactions, contributed to the intense binding force between the ligand SNX-2112 and its target Hsp90^*N*^. SNX-2112 formed three hydrogen bonds with residue Y139 (2.7 Å), residue D93 (2.9 Å), and residue K58 (3.0 Å) of Hsp90^*N*^. Meanwhile, SNX-2112 also arranged a network of water-mediated hydrogen bonds with residues S52, D93, and T184. The special feature of SNX-2112 contained three fluorine atoms, and one of them formed a halogen bond (2.7 Å) with the carbonyl group on the main chain of residue G135. In addition, the side chain of residue F138 faced a parallel plan with the pyrazol ring of SNX-2112, for which π-π interaction contributed to their binding. SNX-2112 formed multiple hydrophobic interactions with residues M98, L103, L107, F138, Y139, W162, N51, A55, D93, and T184 of Hsp90^*N*^, which was responsible for the intense binding force between the ligand SNX-2112 and its target Hsp90^*N*^.

### New SNX-2112 Derivatives Design and Molecular Docking Evaluation

#### New SNX-2112 Derivatives Design

**FIGURE 6:**
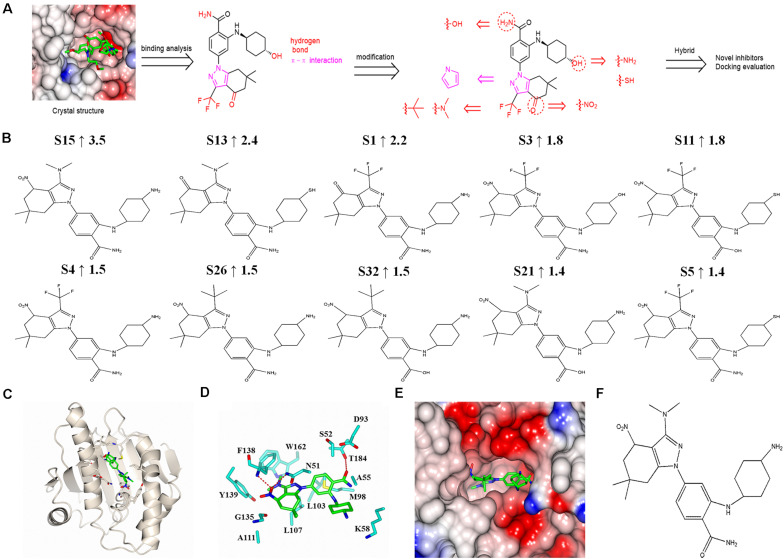
Design and molecular docking evaluation results of new SNX-2112 derivatives. **(A)** Design of new SNX-2112 derivatives based on complex crystal structure and molecular interaction analysis of Hsp90^*N*^-SNX-2112. **(B)** Top 10 optimal new SNX-2112 derivatives with high total score increment evaluated by molecular docking using software SYBYL-X 2.0. **(C)** Overall fold of complex crystal of Hsp90^*N*^-S15. Hsp90^*N*^ and S15 are shown as cartoon and stick, respectively. **(D)** Molecular interaction between Hsp90^*N*^ and S15. The carbon, nitrogen, and oxygen atoms of S15 are colored in green, blue, and red, respectively. In addition, the carbon, nitrogen, oxygen, and sulfur atoms of Hsp90^*N*^ residues are shown in cyan, blue, red, and yellow, respectively. The hydrogen bonds are represented as red dashed lines. **(E)** Connolly surface of ATP-binding site of S15 in Hsp90^*N*^. **(F)** Molecular structure of S15.

Based on the complex crystal structure and molecular interaction analysis, 32 new SNX-2112 derivatives were designed, as shown in Figure 6A. It was important to maintain the special molecular configuration to achieve further structural optimization of SNX-2112. Hydrogen bonds were favorable binding force, and thus, hydrophilic groups were adopted for structural modification of SNX-2112. For instance, the hydroxy group on cyclohexanol moiety was varied to an amino group or sulfhydryl group. The amino group on benzamide moiety was replaced by a hydroxy group. The carbonyl group of 6,6-dimethyl-1,5,6,7-tetrahydro-indazol-4-one moiety was substituted by a nitro group. Hydrophobic interactions also contributed more, and introduction of hydrophobic functional groups would be a suitable scheme for structural modification. To be specific, the trifluoromethyl part was modified to a dimethylamino or tert-butyl group. Considering the π-π interactions formed, the ^1^*H*-pyrazole ring of SNX-2112 was replaced by a ^1^*H*-pyrrole ring.

#### Molecular Docking Evaluation

Based on the design scheme, 32 new SNX-2112 derivatives were designed, and the binding capacity with the target Hsp90^*N*^ was evaluated by molecular docking from theoretical calculation aspect using the software SYBYL-X 2.0. Total score and CScore were chosen as key evaluation parameters, which were positively related to their binding force. Their chemical structures, docking results, and total score increment compared with SNX-2112 are shown in Supplementary Table 1. Among them, a total score of 25 new derivatives increased compared with SNX-2112, which suggested a favorable design scheme. The top 10 new derivatives with high total score increment compared with SNX-2112 and chemical structures were shown in Figure 6B. The total score of the optimal one S15 was as high as 9.5 (increment, 3.5 vs. SNX-2112), which suggested that there was an intense banding force between S15 and its target Hsp90^*N*^, and S15 was a favorable ligand for target Hsp90^*N*^. There were obvious differences in structures between S15 and SNX-2112. First, compared to SNX-2112, the hydroxy group on cyclohexanol moiety of SNX-2112 was varied to an amino group in S15. Second, the carbonyl group of 6,6-dimethyl-1,5,6,7-tetrahydro-indazol-4-one moiety of SNX-2112 was substituted by a nitro group in S15. Last, the trifluoromethyl part of SNX-2112 was modified to a dimethylamino group in S15. The changes in the structure improved the Hsp90^*N*^-S15 binding affinity.

In the optimal derivative S15 as a representative (Figure 6F), simulated complex 3D structure reconstruction and molecular interaction analysis of Hsp90^*N*^-S15 were performed by SYBYL software and CCP4MG software, as shown in Figures 6C–E. S15 was suitably located in the ATP-binding pocket of Hsp90^*N*^ (Figures 6C,E). Hydrogen bonds were responsible for their intense binding force. S15 formed three hydrogen bonds with residues N51 (2.9 Å), F138 (2.7 Å), and T184 (3.0 Å) of Hsp90^*N*^. Moreover, the hydrophobic effects apparently also played a vital role for binding of S15 to Hsp90^*N*^. Specifically, the hydrophobic interactions appeared between the 4,5,6,7-tetrahydro-^1^*H*-indazole moiety and residues L107, G135, and Y139 of Hsp90^*N*^. In addition, the benzamide moiety together with cyclohexylamine moiety formed the hydrophobic interactions with residues A55, K58, and M98 (Figure 6D).

In short, hydrogen bonds and hydrophobic interactions of Hsp90^*N*^-S15 were responsible for their intense binding force, which would provide a potential lead compound for anti-NSCLC new drug development.

## Discussion

Non–small cell lung cancer accounts for more than 80% of lung cancer cases, which is the leading cause of cancer death worldwide (Petrek and Yu, 2019). Targeted drugs, for NSCLC as an oncogene addicted tumor, could be an effective way of treatment. However, drug resistance and toxicity confined their clinical applications (Tomasini et al., 2016; Xiong et al., 2019). Hsp90, as a chaperone, plays a vital and even decisive role in tumorigenesis and development by assisting 300 client proteins to obtain their correct folding and mature conformation (Jhaveri et al., 2014; Hyun et al., 2018). Hsp90 exists with an α-α or β-β homodimer in the cytoplasm, and each monomer consists of three domains. The C-terminal domain (Hsp90^*C*^) plays a key role for dimerization of Hsp90, whereas the central domain (Hsp90^*M*^) possesses a large hydrophobic surface to bind client proteins and facilitate their folding. The *N*-terminal domain (Hsp90^*N*^), with ATP-hydrolase activity, contains an ATP-binding pocket that is responsible for ATP binding and hydrolysis to provide energy for client protein correct folding or conformation maturation, which is indispensable for the chaperone function of Hsp90 (Donnelly and Blagg, 2008; Mayer et al., 2012).

Accordingly, Hsp90 inhibitors were divided as Hsp90^*N*^ inhibitors, Hsp90^*M*^ inhibitors and Hsp90^*C*^ inhibitors. Hsp90^*N*^ inhibitors could bind in the ATP-binding pocket to prevent ATP binding and then disable chaperone function of Hsp90 (Prodromou et al., 2000; Li et al., 2012b). Hsp90^*N*^ inhibitor SNX-2112 was perfectly sitting at the ATP-binding pocket of Hsp90^*N*^, which prevented ATP binding to disable chaperone function of Hsp90. Therefore, immature or misfolding client proteins would be captured and degraded by proteasomes (Chen et al., 2014). With degraded proteins accumulated, multiple signaling pathways can be derailed simultaneously, which can hinder proliferation, and progression of cancer (Chehab et al., 2015; Dutta Gupta et al., 2019). Hsp90, therefore, was paid more attention to as a promising anticancer drug target (Barrott and Haystead, 2013; Soga et al., 2013; Hu et al., 2019).

Absence of the complex crystal structure of Hsp90^*N*^-SNX-2112, however, prevented further structural optimization of SNX-2112 and understanding of binding mode and molecular interaction of Hsp90^*N*^-SNX-2112. A high-resolution complex crystal structure of Hsp90^*N*^-SNX-2112 was successfully determined by XRD (resolution limit, 2.10 Å, PDB ID 6LTK), and their molecular interaction was analyzed in detail. The complex crystal structure suggested that SNX-2112 was well accommodated in the ATP-binding pocket to disable molecular chaperone activity of Hsp90, therefore exhibiting favorable inhibiting activity on three NSCLC cell lines (IC_50_, 0.50 ± 0.01 μM for A549, 1.14 ± 1.11 μM for H1299, and 2.36 ± 0.82 μM for H1975) by inhibited proliferation, induced cell cycle arrest, and aggravated cell apoptosis.

The molecular binding mode and interaction mechanism of Hsp90^*N*^-SNX-2112 were analyzed in detail by the complex crystal structure, which suggested that hydrogen bonds, hydrophobic interactions, and π-π interactions significantly contributed to the intense binding force between the ligand SNX-2112 and its target Hsp90^*N*^, which was verified by TSA and ITC. TSA was a useful tool for target-ligand binding force valuation by determining melting temperature differences (ΔTm) of the target with or without binding a ligand. Every protein has a definite melting temperature under certain conditions, and it changes after binding a ligand. Greater absolute ΔTm value means stronger binding force. It is deemed as a favorable ligand with absolute ΔTm value greater than 3. The TSA result (ΔTm, -9.51 ± 1.00°C) indicated an intense binding force between Hsp90^*N*^ and SNX-2112, which was verified again by ITC. ITC, another tool for binding force determination, measured heat absorption or release amount during target-ligand binding process. Smaller *K*_*d*_ means stronger binding force and protein thermostability. The ITC results (*K*_*d*_, 14.10 ± 1.60 nM) suggested that the thermostability of Hsp90^*N*^ increased binding with its ligand SNX-2112.

Based on the complex crystal structure, molecular interaction analysis from TSA and ITC, the design scheme of new SNX-2112 derivatives focused on promoting the interactions by group replacements, such as hydrogen bonds, hydrophobic interactions, and π-π interactions, contributed to the intense binding force of Hsp90^*N*^-SNX-2112. Thirty-two novel SNX-2112 derivatives were designed, and 25 exhibited increased binding force with the target Hsp90^*N*^, which confirmed a favorable design scheme evaluated by molecular docking assay. The results would provide promising lead compounds for anti-NSCLC new drug development based on the lead compound SNX-2112.

## Conclusion

SNX-2112 is a promising compound, but the absence of the complex crystal structure of Hsp90N-SNX-2112 restricted the structural optimization of SNX-2112. Herein, a complex crystal structure of Hsp90^*N*^-SNX-2112 was determined, and anti-NSCLC activity *in vitro* of SNX-2112 was evaluated. The main results were as follows:

(1) SNX-2112 displayed favorable anti-NSCLC activity (IC_50_, 0.50 ± 0.01 μM for A549, 1.14 ± 1.11 μM for H1299, and 2.36 ± 0.82 μM for H1975) by inhibiting cell viability, inducing cell cycle arrest, and aggravating cell apoptosis.
(2) A high-resolution complex crystal structure Hsp90^*N*^-SNX-2112 was successfully determined by XRD (resolution limit, 2.14 Å, PDB ID 6LTK). It was indicated that SNX-2112 well accommodated in the ATP-binding pocket to disable molecular chaperone function of Hsp90, therefore suppressing cancer cells.
(3) The result from TSA disclosed that an intense binding force resulted in a more stable conformation of Hsp90^*N*^ after binding with SNX-2112 (ΔTm, -9.51 ± 1.00°C), which was verified from favorable thermodynamic changes by ITC (*K*_*d*_, 14.10 ± 1.60 nM).
(4) Based on complex crystal structure and molecular interaction analysis of Hsp90^*N*^-SNX-2112, 32 new SNX-2112 derivatives were successfully designed. Among them, 25 new derivatives exhibited increased binding force with Hsp90^*N*^, which verified a favorable design scheme by molecular docking evaluation.
(5) The results would provide new references and guides for anti-NSCLC new drug development basis on the lead compound SNX-2112.

## Data Availability Statement

The datasets presented in this study can be found in online repositories. The names of the repository/repositories and accession number(s) can be found in the article/Supplementary Material.

## Author Contributions

DZ, Y-MX, and L-QC performed the experiments, analyzed the data, interpreted the results, and wrote the manuscript. FY, HZ, and WQ collected and determined the data. H-JL, C-XH, and XJ participated in the cell study. LX, XZ, and P-QL participated in the molecular interaction analysis. YH, J-HH, and H-LC participated in the study design, result interpretation and manuscript improvement. All authors read and approved the final manuscript.

## Conflict of Interest

The authors declare that the research was conducted in the absence of any commercial or financial relationships that could be construed as a potential conflict of interest.

## Supplementary Material

Supplementary Image 1 Complex crystals of Hsp90^*N*^-SNX-2112. Complex crystals were obtained by the hanging-drop method at 4°C for 3–5 days. The average dimension of rhombus crystals was approximately 230 μm × 130 μm × 50 μm.

Supplementary Table 1 Results for molecular docking evaluation of SNX-2112 or its new derivatives binding with the target Hsp90^*N*^.

## References

[B1] Adams P. Afonine P. Bunkóczi G. Chen V. Davis I. Echols N. (2010). PHENIX: a comprehensive python-based system for macromolecular structure solution. *Acta Crystallogr. Sect D: Biol. Crystallogr.* 66 213–221. 10.1107/s0907444909052925 20124702 PMC2815670

[B2] Andreotti G. Monticelli M. Cubellis M. (2015). Looking for protein stabilizing drugs with thermal shift assay. *Drug Test. Anal.* 7 831–834. 10.1002/dta.1798 25845367 PMC6681132

[B3] Bachleitner-Hofmann T. Sun M. Chen C. Liska D. Zeng Z. Viale A. (2011). Antitumor activity of SNX-2112, a synthetic heat shock protein-90 inhibitor, in MET-amplified tumor cells with or without resistance to selective MET Inhibition. *Clin. Cancer Res.* 17 122–133. 10.1158/1078-0432.ccr-10-0253 21208906 PMC3263825

[B4] Barrott J. Haystead T. (2013). Hsp90, an unlikely ally in the war on cancer. *FEBS J.* 280 1381–1396. 10.1111/febs.12147 23356585 PMC3815692

[B5] Cao H. L. Lyu K. K. Liu B. Li J. He J. H. (2017). Discovery of a novel small inhibitor RJ19 targeting to human Hsp90. *Nucl. Sci. Tech.* 28:148. 10.1007/s41365-017-0300-1

[B6] Chehab M. Caza T. Skotnicki K. Landas S. Bratslavsky G. Mollapour M. (2015). Targeting Hsp90 in urothelial carcinoma. *Oncotarget* 6 8454–8473. 10.18632/oncotarget.3502 25909217 PMC4496161

[B7] Chen D. Shen A. Li J. Shi F. Chen W. Ren J. (2014). Discovery of potent N-(isoxazol-5-yl)amides as HSP90 inhibitors. *Eur. J. Med. Chem.* 87 765–781. 10.1016/j.ejmech.2014.09.065 25313505

[B8] Donnelly A. Blagg B. (2008). Novobiocin and additional inhibitors of the Hsp90 C-terminal nucleotide-binding pocket. *Curr. Med. Chem.* 15 2702–2717. 10.2174/092986708786242895 18991631 PMC2729083

[B9] Dutta Gupta S. Bommaka M. Banerjee A. (2019). Inhibiting protein-protein interactions of Hsp90 as a novel approach for targeting cancer. *Eur. J. Med. Chem.* 178 48–63. 10.1016/j.ejmech.2019.05.073 31176095

[B10] Emsley P. Lohkamp B. Scott W. Cowtan K. (2010). Features and development of coot. *Acta Crystallogr. Sect D: Biol. Crystallogr.* 66 486–501. 10.1107/s0907444910007493 20383002 PMC2852313

[B11] Ettinger D. Wood D. Aisner D. Akerley W. Bauman J. Chirieac L. (2017). Non-small cell lung cancer, version 5.2017, NCCN clinical practice guidelines in oncology. *J. Natl. Compr. Canc. Ne.* 15 504–535. 10.6004/jnccn.2017.0050 28404761

[B12] Fernandes J. Alves P. (2017). Recent patents on heat shock proteins targeting antibodies. *Recent Pat. Anticancer Drug Disco.* 12 48–54. 10.2174/1574892812666161123141516 27881057

[B13] He Y. Bubb A. Stubbs K. Gloster T. Davies G. (2011). Inhibition of a bacterial O-GlcNAcase homologue by lactone and lactam derivatives: structural, kinetic and thermodynamic analyses. *Amino Acids* 40 829–839. 10.1007/s00726-010-0700-6 20689974

[B14] He Y. Hao Q. Li W. Yan C. Yan N. Yin P. (2014a). Identification and characterization of ABA receptors in *Oryza sativa*. *PLoS One* 9:e95246. 10.1371/journal.pone.0095246 24743650 PMC3990689

[B15] He Y. Roth C. Turkenburg J. Davies G. (2014b). Three-dimensional structure of a *Streptomyces sviceus* GNAT acetyltransferase with similarity to the C-terminal domain of the human GH84 O-GlcNAcase. *Acta Crystallogr. Sect D: Biol. Crystallogr.* 70 186–195. 10.1107/s1399004713029155 24419391 PMC3919268

[B16] Hendriks L. Dingemans A. (2017). Heat shock protein antagonists in early stage clinical trials for NSCLC. *Expert Opin. Inv. Drug* 26 541–550. 10.1080/13543784.2017.1302428 28274158

[B17] Hu L. Wang Y. Chen Z. Fu L. Wang S. Zhang X. (2019). Hsp90 Inhibitor SNX-2112 enhances TRAIL-Induced apoptosis of human cervical cancer cells via the ROS-Mediated JNK-p53-Autophagy-DR5 Pathway. *Oxid. Med. Cell. Longev.* 2019:9675450. 10.1155/2019/9675450 31019655 PMC6452544

[B18] Huang T. Chu H. Lin Y. Ho W. Hou H. Chao Y. (2009). Minocycline attenuates 5-fluorouracil-induced small intestinal mucositis in mouse model. *Biochem. Bioph. Res. Co.* 389 634–639. 10.1016/j.bbrc.2009.09.041 19765544

[B19] Hyun S. Le H. Nguyen C. Yong Y. Boo H. Lee H. (2018). Development of a novel Hsp90 inhibitor NCT-50 as a potential anticancer agent for the treatment of non-small cell lung cancer. *Sci. Rep.* 8:13924. 10.1038/s41598-018-32196-6 30224681 PMC6141536

[B20] Jhaveri K. Ochiana S. Dunphy M. Gerecitano J. Corben A. Peter R. (2014). Heat shock protein 90 inhibitors in the treatment of cancer: current status and future directions. *Expert Opin. Inv. Drug* 23 611–628. 10.1517/13543784.2014.902442 24669860 PMC4161020

[B21] Li J. Shi F. Chen D. Q. Cao H. L. Xiong B. Shen J. K. (2015). FS23 binds to the N-terminal domain of human Hsp90: a novel small inhibitor for Hsp90. *Nucl. Sci. Tech.* 26:060503. 10.13538/j.1001-8042/nst.26.060503

[B22] Li J. Soroka J. Buchner J. (2012a). The Hsp90 chaperone machinery: conformational dynamics and regulation by co-chaperones. *Biochim. Biophys. Acta* 1823 624–635. 10.1016/j.bbamcr.2011.09.003 21951723

[B23] Li J. Sun L. Xu C. Yu F. Zhou H. Zhao Y. (2012b). Structure insights into mechanisms of ATP hydrolysis and the activation of human heat-shock protein 90. *Acta Bioch. Bioph. Sin.* 44 300–306. 10.1093/abbs/gms001 22318716

[B24] Lo M. Aulabaugh A. Jin G. Cowling R. Bard J. Malamas M. (2004). Evaluation of fluorescence-based thermal shift assays for hit identification in drug discovery. *Anal. Biochem.* 332 153–159. 10.1016/j.ab.2004.04.031 15301960

[B25] Mayer P. Harjung A. Breinig M. Fischer L. Ehemann V. Malz M. (2012). Expression and therapeutic relevance of heat-shock protein 90 in pancreatic endocrine tumors. *Endocrine-related cancer* 19 217–232. 10.1530/erc-11-0227 22194440

[B26] McNicholas S. Potterton E. Wilson K. Noble M. (2011). Presenting your structures: the CCP4mg molecular-graphics software. *Acta Crystallogr., Sect D: Biol. Crystallogr.* 67 386–394. 10.1107/s0907444911007281 21460457 PMC3069754

[B27] Molina J. Yang P. Cassivi S. Schild S. Adjei A. (2008). Non-small cell lung cancer: epidemiology, risk factors, treatment, and survivorship. *Mayo Clin. Proc.* 83 584–594. 10.4065/83.5.584 18452692 PMC2718421

[B28] Mollapour M. Tsutsumi S. Kim Y. Trepel J. Neckers L. (2011). Casein kinase 2 phosphorylation of Hsp90 threonine 22 modulates chaperone function and drug sensitivity. *Oncotarget* 2 407–417. 10.18632/oncotarget.272 21576760 PMC3248188

[B29] Okawa Y. Hideshima T. Steed P. Vallet S. Hall S. Huang K. (2009). SNX-2112, a selective Hsp90 inhibitor, potently inhibits tumor cell growth, angiogenesis, and osteoclastogenesis in multiple myeloma and other hematologic tumors by abrogating signaling via Akt and ERK. *Blood* 113 846–855. 10.1182/blood-2008-04-151928 18948577 PMC2630270

[B30] Olszewska P. Cal D. Zagórski P. Mikiciuk-Olasik E. (2020). A novel trifluoromethyl 2-phosphonopyrrole analogue inhibits human cancer cell migration and growth by cell cycle arrest at G1 phase and apoptosis. *Eur. J. Pharmacol.* 871:172943. 10.1016/j.ejphar.2020.172943 31978423

[B31] Petrek H. Yu A. (2019). MicroRNAs in non-small cell lung cancer: Gene regulation, impact on cancer cellular processes, and therapeutic potential. *Pharmacol. Res. Persp.* 7:e00528. 10.1002/prp2.528 31859460 PMC6923806

[B32] Prodromou C. Panaretou B. Chohan S. Siligardi G. O’Brien R. Ladbury J. (2000). The ATPase cycle of Hsp90 drives a molecular ‘clamp’ via transient dimerization of the N-terminal domains. *EMBO J.* 19 4383–4392. 10.1093/emboj/19.16.4383 10944121 PMC302038

[B33] Sidera K. Patsavoudi E. (2014). HSP90 inhibitors: current development and potential in cancer therapy. *Recent Pat. Anticancer Drug Disco.* 9 1–20. 10.2174/157488980901140428100532 23312026

[B34] Soga S. Akinaga S. Shiotsu Y. (2013). Hsp90 inhibitors as anti-cancer agents, from basic discoveries to clinical development. *Curr. Pharm. Design* 19 366–376. 10.2174/138161213804143617 22920907

[B35] Stebbins C. Russo A. Schneider C. Rosen N. Hartl F. Pavletich N. (1997). Crystal structure of an Hsp90-geldanamycin complex: targeting of a protein chaperone by an antitumor agent. *Cell* 89 239–250. 10.1016/s0092-8674(00)80203-2 9108479

[B36] Sung N. Lee J. Kim J. Chang C. Joachimiak A. Lee S. (2016). Mitochondrial Hsp90 is a ligand-activated molecular chaperone coupling ATP binding to dimer closure through a coiled-coil intermediate. *Proc. Nati. Acad. Sci. U S A.* 113 2952–2957. 10.1073/pnas.1516167113 26929380 PMC4801263

[B37] Tabata M. Tsubaki M. Takeda T. Tateishi K. Maekawa S. Tsurushima K. (2020). Inhibition of HSP90 overcomes melphalan resistance through downregulation of Src in multiple myeloma cells. *Clin. Exp. Med.* 20 63–71. 10.1007/s10238-019-00587-2 31650359

[B38] Tomasini P. Walia P. Labbe C. Jao K. Leighl N. (2016). Targeting the KRAS pathway in non-small cell lung cancer. *Oncologist* 21 1450–1460. 10.1634/theoncologist.2015-0084 27807303 PMC5153335

[B39] Vahid S. Thaper D. Zoubeidi A. (2017). Chaperoning the Cancer: the proteostatic functions of the heat shock proteins in Cancer. *Recent Pat. Anticancer Drug Disco.* 12 35–47. 10.2174/1574892811666161102125252 27809750

[B40] Wang Q. S. Yu F. Huang S. Sun B. Zhang K. H. Liu K. (2015). The macromolecular crystallography beamline of SSRF. *Nucl. Sci. Tech.* 26:010102. 10.13538/j.1001-8042/nst.26.010102

[B41] Wang Q. S. Zhang K. H. Cui Y. Wang Z. J. Pan Q. Y. Liu K. (2018). Upgrade of macromolecular crystallography beamline BL17U1 at SSRF. *Nucl. Sci. Tech.* 29:68. 10.1007/s41365-018-0398-9

[B42] Wang X. Wang S. Liu Y. Ding W. Zheng K. Xiang Y. (2014). The Hsp90 inhibitor SNX-2112 induces apoptosis of human hepatocellular carcinoma cells: the role of ER stress. *Biochem. Bioph. Res. Co.* 446 160–166. 10.1016/j.bbrc.2014.02.081 24582562

[B43] Wei Q. Ning J. Dai X. Gao Y. Su L. Zhao B. (2018). Discovery of novel HSP90 inhibitors that induced apoptosis and impaired autophagic flux in A549 lung cancer cells. *Eur. J. Med. Chem.* 145 551–558. 10.1016/j.ejmech.2018.01.024 29339250

[B44] Winn M. Ballard C. Cowtan K. Dodson E. Emsley P. Evans P. (2011). Overview of the CCP4 suite and current developments. *Acta Crystallogr., Sect D: Biol. Crystallogr.* 67 235–242. 10.1107/s0907444910045749 21460441 PMC3069738

[B45] Xiong Y. He L. Shay C. Lang L. Loveless J. Yu J. (2019). Nck-associated protein 1 associates with HSP90 to drive metastasis in human non-small-cell lung cancer. *J. Exp. Clin. Cancer Res.* 38:122. 10.1186/s13046-019-1124-0 30867003 PMC6417146

[B46] Xue M. Pang H. Li X. Li H. Pan J. Chen W. (2016). Long non-coding RNA urothelial cancer-associated 1 promotes bladder cancer cell migration and invasion by way of the hsa-miR-145-ZEB1/2-FSCN1 pathway. *Cancer Sci.* 107 18–27. 10.1111/cas.12844 26544536 PMC4724815

[B47] Ye K. Wei Q. Gong Z. Huang Y. Liu H. Li Y. (2017). Effect of norcantharidin on the proliferation, apoptosis, and cell cycle of human mesangial cells. *Renal Failure* 39 458–464. 10.1080/0886022x.2017.1308257 28393592 PMC6014529

[B48] Yu F. Wang Q. Li M. Zhou H. Liu K. Zhang K. (2019a). Aquarium: an automatic data-processing and experiment information management system for biological macromolecular crystallography beamlines. *J. Appl. Crystallogr.* 52 472–477. 10.1107/S1600576719001183

[B49] Yu Z. Zheng C. Chen G. Huang R. Zhou X. Niu Z. (2019b). 3,4- seco-norclerodane diterpenoids from the roots of *Polyalthia laui*. *J. Nat. Prod.* 82 27–34. 10.1021/acs.jnatprod.8b00243 30596489

