# Supplementary figures and images for: Complex Crystal Structure Determination and in vitro Anti–non–small Cell Lung Cancer Activity of Hsp90N Inhibitor SNX-2112
Source: Front Cell Dev Biol. 2021 Mar 29;9:650106. doi: 10.3389/fcell.2021.650106 (PMC8039390; doi:10.3389/fcell.2021.650106)

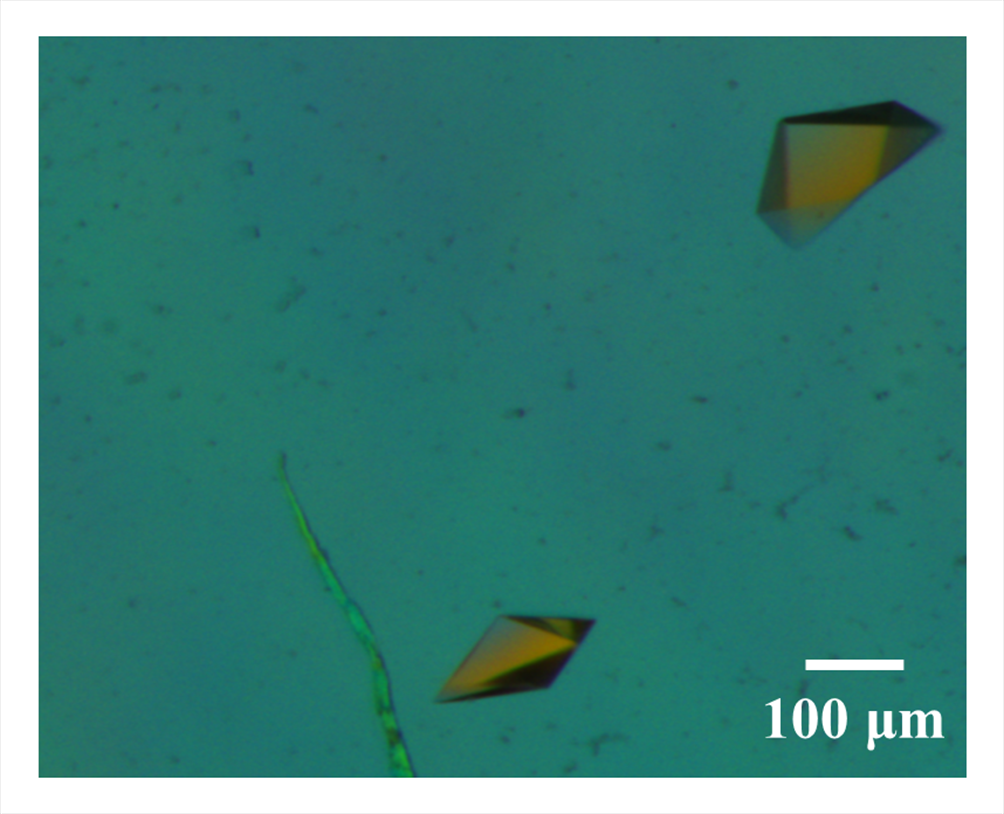

Supplement: Supplementary Image 1 — Complex crystals of Hsp90N-SNX-2112. Complex crystals were obtained by the hanging-drop method at 4°C for 3–5 days. The average dimension of rhombus crystals was approximately 230 μm × 130 μm × 50 μm. [file Image_1.tif]
