# Supplementary material for: Complex Crystal Structure Determination and in vitro Anti–non–small Cell Lung Cancer Activity of Hsp90N Inhibitor SNX-2112
Source: Front Cell Dev Biol. 2021 Mar 29;9:650106. doi: 10.3389/fcell.2021.650106 (PMC8039390; doi:10.3389/fcell.2021.650106)
Supplement: Supplementary Table 1 — Results for molecular docking evaluation of SNX-2112 or its new derivatives binding with the target Hsp90N. [file Table_1.docx]

**Table A. Results for molecular docking evaluation of SNX-2112 and its new derivatives**

| **Name** | **Total Score** | **CScore** | **Molecular structure** | **Total Score**  **increment** |
| --- | --- | --- | --- | --- |
| **S** | 6.0 | 1 | 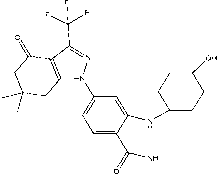 |  |
| **S15** | 9.5 | 4 | 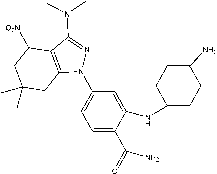 | ↑3.5 |
| **S13** | 8.4 | 4 | 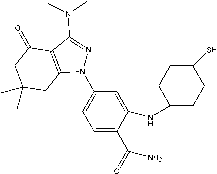 | ↑2.4 |
| **S1** | 8.2 | 3 | 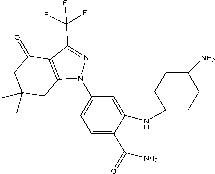 | ↑2.2 |
| **S3** | 7.8 | 2 | 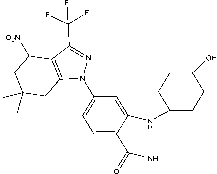 | ↑1.8 |
| **S11** | 7.8 | 2 | 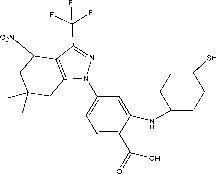 | ↑1.8 |
| **S4** | 7.5 | 2 | 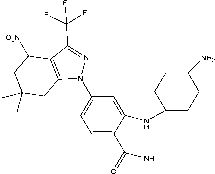 | ↑1.5 |
| **S26** | 7.5 | 2 | 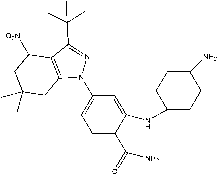 | ↑1.5 |
| **S32** | 7.5 | 2 | 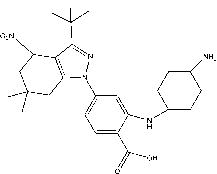 | ↑1.5 |
| **S21** | 7.4 | 1 | 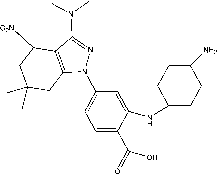 | ↑1.4 |
| **S5** | 7.4 | 1 | 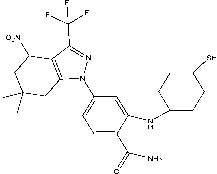 | ↑1.4 |
| **S17** | 7.2 | 2 | 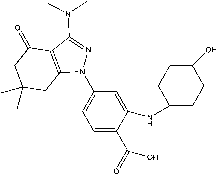 | ↑1.2 |
| **S29** | 7.1 | 2 | 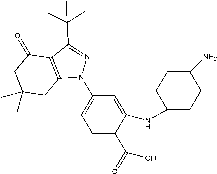 | ↑1.1 |
| **S7** | 6.8 | 2 | 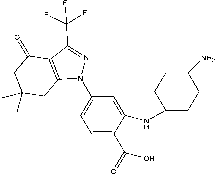 | ↑0.8 |
| **S20** | 6.8 | 2 | 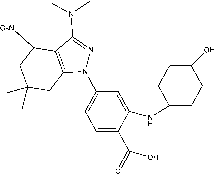 | ↑0.8 |
| **S19** | 6.8 | 2 | 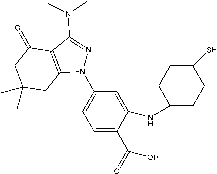 | ↑0.8 |
| **S10** | 6.7 | 2 | 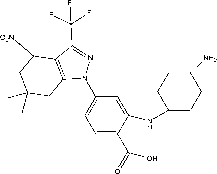 | ↑0.7 |
| **S18** | 6.6 | 2 | 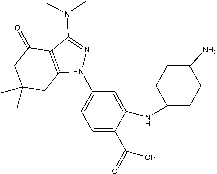 | ↑0.6 |
| **S6** | 6.5 | 2 | 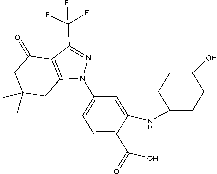 | ↑0.5 |
| **S8** | 6.5 | 1 | 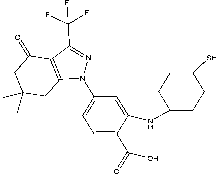 | ↑0.5 |
| **S2** | 6.4 | 2 | 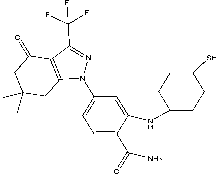 | ↑0.4 |
| **S28** | 6.4 | 1 | 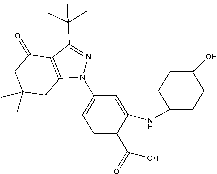 | ↑0.4 |
| **S14** | 6.2 | 3 | 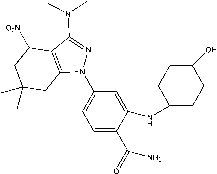 | ↑0.2 |
| **S9** | 6.2 | 1 | 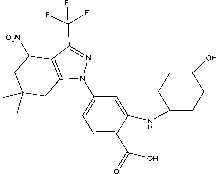 | ↑0.2 |
| **S25** | 6.2 | 3 | 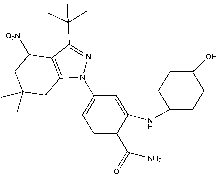 | ↑0.2 |
| **S30** | 6.1 | 1 | 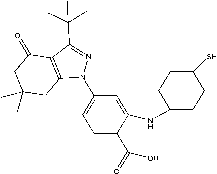 | ↑0.1 |
| **S33** | 5.9 | 2 | 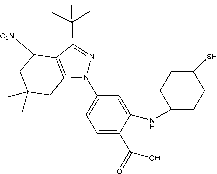 | ↓0.1 |
| **S16** | 5.9 | 3 | 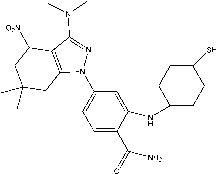 | ↓0.1 |
| **S27** | 5.8 | 2 | 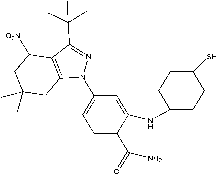 | ↓0.2 |
| **S24** | 5.7 | 1 | 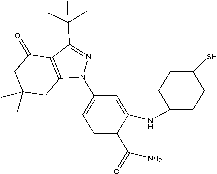 | ↓0.3 |
| **S31** | 5.6 | 2 | 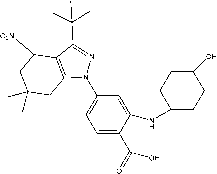 | ↓0.4 |
| **S22** | 5.5 | 2 | 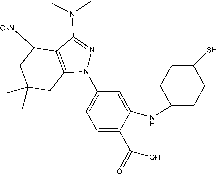 | ↓0.5 |
| **S12** | 5.4 | 2 | 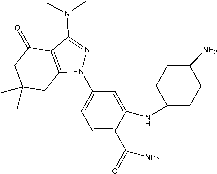 | ↓0.6 |
